# Contemporary Understanding of the Etiology and Management of Molar Incisor Hypomineralization: A Literature Review

**DOI:** 10.3390/dj11070157

**Published:** 2023-06-23

**Authors:** Ahmed Yahya Alzahrani, Najlaa Mohammed Hussain Alamoudi, Omar Abd El Sadek El Meligy

**Affiliations:** 1 Pediatric Dentistry Department, Faculty of Dentistry, King Abdulaziz University, P.O. Box 80209, Jeddah 21589, Saudi Arabia; aalzahrani3670@stu.kau.edu.sa (A.Y.A.); nalamoudi@kau.edu.sa (N.M.H.A.); 2 Pediatric Dentistry, Taif Dental Centre, Ministry of Health, Taif 26511, Saudi Arabia; 3 Pediatric Dentistry & Dental Public Health Department, Faculty of Dentistry, Alexandria University, Alexandria 21521, Egypt

**Keywords:** molar hypomineralization, etiology, treatment, child, adolescent, review

## Abstract

Molar incisor hypomineralization (MIH) is a significant health problem that can affect the child’s quality of life by negatively affecting their esthetics and function. This review aimed to summarize the etiology and pathogenesis of MIH. It also aimed to summarize the recent studies on MIH in children and adolescents, focusing on diagnosis, prevention, and clinical management. An electronic search on the PubMed, Cochrane Database of Systematic Reviews, MEDLINE, MedlinePlus, WHO reports, and Google Scholar databases was performed. The volume of research on the etiology, presentation, and clinical management of MIH is still expanding. The creation and validation of indices for the diagnosis and management of MIH, as well as any potential genetic aspects appear to be the main areas of current research. Notably, MIH was linked to childhood illnesses, the use of antibiotics, and early childhood fever. Although many studies discuss the different options for managing MIH lesions, evidence-based studies that address the long-term outcomes of MIH are still lacking. Indeed, future clinical studies should be directed to evaluate the impact of each systemic etiological factor and its different types of management on normal amelogenesis. Regarding the diagnosis, future research should focus on the pre-eruption diagnosis and early approaches to prevent the post-eruption breakdown and caries. Regarding the treatment of MIH, future investigations should emphasize further improvements in adhesion and the use of new materials and techniques, such as digital dentistry.

## 1. Introduction

Molar incisor hypomineralization (MIH) is defined as a developmental, qualitative enamel defect caused by reduced mineralization and inorganic enamel components, leading to enamel discoloration and fractures of the affected teeth [1]. Enamel hypoplasia can be defined as a defect in the quantity of enamel that is mainly due to disorders in the process of amelogenesis (enamel formation) at the phase of enamel matrix secretion. On the other hand, enamel hypomineralization can be defined as a defect in the enamel quality that is mainly due to the manifestation of disorders in either the maturation or calcification of enamel [1,2]. 

MIH is a qualitative defect affecting the enamel of the first permanent molars, with and without the involvement of incisors. The enamel can present clearly demarcated opacities with an alteration in the translucency, showing a wide variation in color, size, and shape [3]. From a histological perspective, MIH is characterized with a reduction in the mineral quantity, specifically in the calcium and phosphate content, resulting in a decrease in the modulus of elasticity and enamel hardness. Furthermore, it exhibits an increase in the protein content accompanied with an apparent porosity in the enamel’s structure. The enamel crystals in MIH have been observed to have a lower density, higher organic content within and between the crystals, and thicker prism sheaths [4,5]. 

The high prevalence and impact of molar incisor hypomineralization (MIH) on children’s oral health has become a major concern in recent years. This condition can have a significant impact on a child’s oral health-related quality of life (OHRQoL), causing pain, functional difficulties, and aesthetic concerns. This makes it challenging to manage in young patients. Furthermore, MIH, the most frequent enamel defect, exhibits a wide range of severity with variation in the clinical presentation [6]. Therefore, from an epidemiologic standpoint, MIH is gaining more attention every year from the community of pediatric dentists due to its global prevalence and negative impact on affected children [7].

Until now, the causes of the disease remained elusive. However, Butera et al. investigated the pre-, peri-, and postnatal factors of this disease, which suggested that issues like genetic and medical complications during pregnancy contribute to MIH, rendering it a multifactorial disease. They advocated for further research involving larger, accurately diagnosed, and ethnically diverse populations to better explore the genetic and environmental factors influencing deciduous molar hypomineralization (DMH), hypomineralized second primary molar (HSPM), and MIH occurrences [8]. This review aimed to encapsulate the modern understanding of MIH etiology and pathogenesis, emphasizing recent studies centered on the diagnosis, prevention, and clinical management for both children and adolescents.

## 2. Methods

### 2.1. Search Strategy and Study Selection

This review article compiles data from published sources discussing MIH between 2013 and 2023. The databases used for extracting articles include PubMed, Cochrane Database of Systematic Reviews, MEDLINE, MedlinePlus, Google Scholar, and WHO reports. We searched for terms relating to MIH and its risk factors, using keywords such as “molar incisor hypomineralization”, “MIH”, “etiology”, “treatment”, “children”, “adolescents”, and “literature review”. Additionally, we manually screened references from our retrieved studies to examine for their potential relevance. The topic was then divided into etiology, diagnosis, and management. All in vitro and in vivo studies within each category were summarized and discussed.

### 2.2. Participants and Eligibility Criteria

The following were the inclusion criteria for the study, which included PICO: (1) patient: MIH youngsters who are under the age of 18. (2) Intervention: we have included research that looked at the causes of MIH and its links to the perinatal, postnatal, and prenatal complications. Furthermore, we have incorporated the studies that examined various management strategies. (3) Participants without any risk exposure or systemic exposure are compared. The authors also incorporated researches on MIH management that used comparison and intervention groups. (4) Outcome indicator: risk variables for the outcome included prenatal factors, such as sickness or infections during pregnancy, perinatal factors, such as birth weight, early delivery, or cesarean section, and postnatal ones, such as pediatric illnesses, antibiotic usage, and high fever [4]. The findings of this research also took MIH treatment plans into account. (5) Study types: we used case-control, cross-sectional, cohort, and clinical trial studies that had been duly registered and authorized by their respective ethical committees. The studies included were all in English.

## 3. Etiology and Pathogenesis of MIH

Although the exact cause of MIH remains unclear, a combination of several factors has been proposed to explain the occurrence of this lesion. Potential causes can be divided into genetic and environmental influences [9]. However, before delving into these factors, it is essential to comprehend how this defect develops, and at which stage of enamel formation can MIH occur.

### 3.1. Amelogenesis Abnormalities in MIH

To reach normal enamel, a standard amelogenesis process must take place. This process can be influenced by several key modulators that need to be integrated at the right time. Both genetic and environmental factors can trigger the formation of abnormal enamel found in MIH. A single factor or a combination of the following can result in MIH: altered secretion of protein by ameloblasts, insufficient proteinases, underdeveloped tight junctions in ameloblasts, programmed cell death of the ameloblasts, reduced bicarbonate formation by ameloblasts, and impaired calcium transportation by the ameloblast [10]. 

The alterations In the secreted proteins by ameloblasts or insufficient proteinases can lead to deficiencies in protein removal. This can then inhibit the enamel’s crystal growth and cause the development of MIH as a result. Additionally, two other mechanisms can impede enamel crystal growth: ameloblast apoptosis (programmed cell death) and underdeveloped ameloblast tight junctions. Both of these mechanisms can result in elevated albumin levels within the enamel matrix, leading to MIH. Furthermore, the acid/base balance is crucial during enamel hydroxyapatite formation, since the crystal growth depends upon a delicate cellular control of the ionic composition and pH of the extracellular fluid. Hydroxyapatite formation during the maturation stage of amelogenesis liberates an enormous quantity of protons. Thus, a sustained crystal growth requires these protons to be neutralized by bicarbonate transported directly into the enamel space [11]. A reduction in the production of bicarbonates by the ameloblasts has been identified as an additional factor in the development of MIH. Similarly, it has also been found that the failed transportation of calcium by the ameloblast can lead to a decrease in the density of minerals inside the enamel, which result in MIH lesions [10]. 

Lastly, it was postulated that for enamel hypomineralization or qualitative abnormality to occur in the enamel, the ameloblasts should be affected during the apposition stage of enamel formation, which is considered as a late stage in the process of enamel formation and maturation [12]. 

### 3.2. Role of Genetics in the Development of MIH

MIH has a complex and multifactorial etiology that is likely to profoundly contribute to the individual’s genetics. It has been estimated that about 20% of MIH can be attributed to the genetic composition of the individual [13]. Moreover, it has been suggested that a gene on chromosome twenty-two near an area termed “SCUBE1” may be responsible for the development of MIH [14]. Other genes were also found to be associated with MIH, including MMP-20, AMBN, ENAM, and AMELX. These genes were found to be responsible for the formation of the enamel. On the other hand, protective genes that prevent the occurrence of MIH were also identified. A gene termed AMELX has since been identified to be protective against MIH, having been found to code for amelogenin formation in the enamel [15]. 

### 3.3. Role of Epigenetics in the Development of MIH

Epigenetics has been defined today as the study of changes in the gene function that are mitotically and/or meiotically heritable, and that do not entail a change in the DNA sequence. Epigenetics is affected by environmental factors, such as the methylation of DNA [16]. The role of epigenetics may explain why the lesion of MIH only affect the occlusal 2/3 of the crown, as epigenetics can cause site-specific lesions by impairing the formation of the enamel at certain stages and sites [10].

### 3.4. Role of Environmental Stressors in the Development of MIH

Numerous studies have examined the association between environmental factors and MIH, reporting similar patterns across several diseases and conditions. For instance, animal experiments have demonstrated that exposure to bisphenol A can result in comparable lesions [17]. Additionally, breast milk contamination with herbicides, such as dioxin, has been linked to enamel defects, including MIH. Furthermore, the elevated prevalence of MIH lesions in urban children, which are twice as prevalent compared to rural children, has been attributed to toxicants associated with industrialization [18]. Although chronic fluoride toxicity was blamed for hypomineralization defects in the enamel due to the toxic effect of excessive fluoride on the ameloblasts, these lesions are different from MIH lesions. Thus, no significant association was found between chronic fluoride toxicity and MIH [6,19].

Possible link factors were investigated to find a relationship between these environmental factors and MIH. Association between problems during pregnancy and MIH has been suggested. In addition, the child’s illnesses, such as pneumonia and asthma, were also found to be associated with MIH. Thus, it was concluded that any of the factors underlying the prenatal causes (such as hypocalcemia and diabetes mellitus), the perinatal causes (such as delivery complications and premature birth), and the postnatal causes (such as poor nutrition and administration of antibiotics) can lead to the development of MIH lesion [20]. 

**Figure 1:**
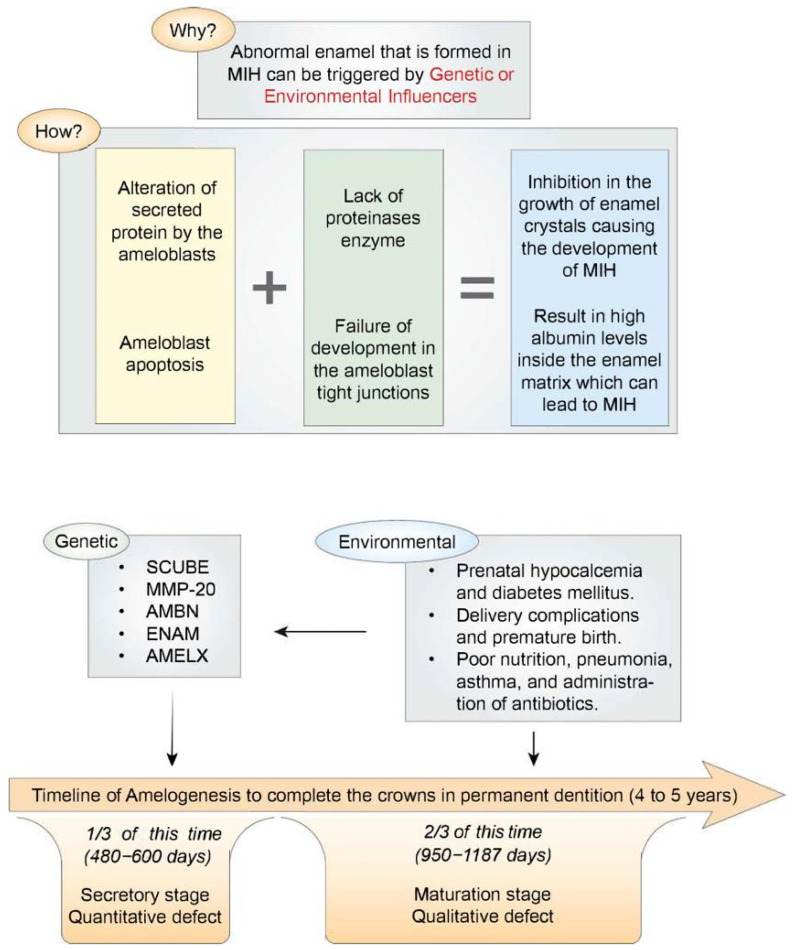
(**Top** part) A schematic illustration summarizing the mechanisms of abnormal enamel development in MIH. (**Bottom** part) A schematic illustration showing the enamel development stages (secretory and maturation stages) arrayed along a timeline running from the beginning of the appositional growth phase (extreme **left**) to the point where ameloblasts regress at the enamel surface (extreme **right**).

Enamel formation is a relatively slow developmental process in general, and this is certainly true for human enamel formation, which can take up to 4–5 years to complete on the crowns of several permanent dentition teeth. Roughly two-thirds of this time, or up to 950–1187 days, is devoted to the amelogenesis maturation stage [21]. Table 1 summarized the potential risk factors that were found to be associated with MIH in the included studies. Figure 1 summarizes the etiological factors and the mechanisms of abnormal enamel development.

## 4. Prevalence of MIH

Concerning the prevalence of MIH in Saudi Arabia, Allazzam et al., reported an MIH prevalence of 8.6% in children attending the dental clinics at King Abdulaziz University (KAU) in Jeddah [14]. Furthermore, Rizk et al. conducted a cross-sectional study in the region of Qassim that included children aged from seven to nine years and found a high prevalence of MIH (25.1%), although the study did not find significant differences in the prevalence of MIH between the upper and lower arches [15].

A recent systematic review describing the prevalence of MIH in the world found that this lesion is considered to have a relatively high prevalence of 13.5%. In addition, more than one-third of the affected children suffered from moderate-to-severe lesions, with the percentage reaching 36.3%. Moreover, the affection of incisors occurred in 36.6% of cases, while the affection of the second primary molars was uncommon in only 3.6% of cases, respectively. The highest continent that suffered from MIH was America, while Asia was found to be the lowest [17].

## 5. Clinical Picture and Diagnostic Criteria of MIH

### 5.1. Clinical Picture of MIH

MIH is usually observed in the form of a change in the enamel translucency with or without discoloration, ranging from yellow to deep brown. The borders of the lesion are usually well-defined. Porosity can be noted in the enamel’s surface making it more susceptible to chipping and leading to the “post–eruptive breakdown of enamel”. This can leave dentin exposed, thereby increasing the susceptibility to caries. MIH in incisors is common on the incisal third, which may cause esthetic problems to the patient [22].

### 5.2. Diagnostic Criteria of MIH

The European Academy of Paediatric Dentistry (EAPD) reinforces the use of specific clinical signs and symptoms to diagnose MIH [23]. The criteria includes the following:

- To diagnose MIH, one to four permanent first molars (FPM) with enamel hypomineralization must be present. The permanent incisors may also be affected simultaneously. A diagnosis of MIH requires the presence of at least one affected FPM, and the severity of the problems increases with the number of involved incisors, molars, and FPMs. Additionally, the second primary molars, premolars, second permanent molars, and canine tips may also exhibit the abnormalities;
- Opacities in the enamel which are well-demarcated with varying colors and sizes;
- Post-eruptive breakdown of the enamel usually occurs after the tooth/teeth eruption;
- Sensitivity in the affected teeth is common, and can range from a slight reaction to outside stimuli to spontaneous hypersensitivity;
- The size and shape of the restorations do not match the conventional caries image. In molars, the restorations extend to the buccal or palatal/lingual smooth surface;
- If one of the first permanent molars is extracted, the other ones should also be examined for opaque areas and atypical restorations. Furthermore, the incisors should be examined for well-demarcated opacities [16].

### 5.3. Severity of MIH

In order to create an effective treatment strategy, the EAPD reinforces the baseline categorization of the abnormalities in MIH as either mild or severe (Table 2).

## 6. Differential Diagnosis 

Differential diagnosis of MIH includes amelogenesis imperfecta, enamel hypoplasia, white spot lesion, and dental fluorosis.

### 6.1. Amelogenesis Imperfecta

Amelogenesis imperfecta is a well-known genetic defect affecting the enamel in both primary and permanent dentition. These lesions result in hypo-mineralized and hypoplastic areas in the enamel, thereby making it difficult to differentiate it from MIH. However, differentiation can be made by noticing the generalized involvement of all teeth and the family history, as this disease is hereditary [24].

### 6.2. Enamel Hypoplasia 

Enamel hypoplasia is a quantitative defect in the enamel characterized with a decreased amount and thickness of the enamel in certain localized areas. Enamel hypoplasia can be observed in rickets and Turner’s hypoplasia. These areas can mimic the post-eruptive breakdown of the enamel seen in MIH. However, the borders of the hypoplastic lesions are smoother and more regular in shape, while in MIH it is more irregular and sharper [25]. 

### 6.3. White Spot Lesion 

White spot lesions are considered as an early stage of the development of dental caries on the tooth’s surface. The cause of these lesions is a prolonged accumulation of biofilm deposits combined with the consumption of fermentable carbohydrates. The main difference between the white spot lesions and MIH is the site of affection. White spot lesions usually occur in the areas susceptible to the stagnation of dental plaque, such as pits and fissures, in addition to the cervical area of the teeth. On the other hand, MIH rarely occurs in these areas [26].

### 6.4. Dental Fluorosis 

Dental fluorosis is defined as an outcome of chronic high fluoride exposure in children, which is a result of the consumption of excessive fluoride during the period of teeth formation. The clinical picture of this lesion is seen as a poorly defined linear hypoplastic patches with variations in color observed ranging from white to brown. The main difference between fluorotic lesions and MIH is in the demarcation of the lesions. In fluorosis, the lesions do not have a clear boundary, while in MIH a distinct boundary exists. History of excessive fluoride consumption can also help in terms of diagnosis [27].

## 7. Approaches for Treatment of MIH 

Prior to initiating the treatment for patients with MIH, it is imperative to establish a comprehensive diagnosis pertaining to the severity and location of MIH lesions. Subsequently, an evidence-based treatment plan can be delineated, accounting for factors such as age, medical history, symptoms, patient/parental cooperation, and severity, as well as patient/parental desires and expectations. The proposed treatment plan could thereby be tailored accordingly [23].

According to William et al., six steps are needed to manage MIH cases, which are as follows:

- Identification of risk;
- Prompt diagnosis;
- Desensitization and remineralization;
- Preventing the occurrence of enamel breakdown and dental caries;
- Restoration of affected teeth or extraction if beyond possible repair;
- Follow-up and maintenance [28].

### 7.1. Hypersensitivity, Remineralization, and Pain Management in MIH 

The significance of early preventive intervention lies in averting enamel breakdown and the incidence of caries. The preventive measures should include instructing both parents and their children on oral hygiene and the methods of caries prevention via home care, such as the use of fluoridated dentifrices. In an office setting, the plan should include the sealing of fissures and pits. The sealant should be examined in regular appointments and replaced if it becomes lost. Furthermore, the professional application of fluoride in gel, foam, or varnish form could be utilized to reduce caries and sensitivity [5,23]. 

Mastication, oral hygiene, and the quality of life can all be affected by hypersensitivity. Treatments for hypersensitivity include 8% arginine and calcium carbonate paste, casein phosphopeptide-amorphous calcium phosphate (CPP-ACP), casein phosphopeptide-amorphous calcium fluoride phosphate (CPP-ACFP), 5% sodium fluoride varnish (with and without tricalcium phosphate), ozone, and low-level laser therapy. All studies that investigated the management of hypersensitivity revealed a reduction after therapy, according to a recent systematic review. However, none of these tactics can be entirely advised due of the moderate-to-high bias risk of the research, short follow-up periods, and small sample sizes [5,23,29].

Remineralization and desensitization by utilizing dentifrices containing bioactive glass have also been found to be successful. A known dentifrice that contains these materials is Novamin. This dentifrice contains very small particles of bioactive glasses used by dental tissues in active repair. Bioactive glasses can remineralize dentin and decrease its permeability, thus treating teeth sensitivity. To enhance the effect of bioactive glass materials, we can apply it in a vacuum-formed clear retainer which the patient can wear during their sleep [7,23,30]. 

A dentifrice containing arginine was also proven to be effective in the management for mild cases of MIH. The ability of arginine to seal the dentinal tubules and block the sensory nerve conduction mediated by the hydrodynamic movement of dentinal fluid was proven. Arginine dentifrices were found to be superior to the well-known desensitizing agents, such as potassium, fluoride, and strontium [31,32,33]. 

A recent study evaluated the desensitizing and remineralizing effect of a new zinc-hydroxyapatite-based paste in sites affected by MIH by assessing the dental sensitivity, tooth wear, and periodontal indexes, and concluded that biomimetic zinc-hydroxyapatite showed a desensitizing effect when used to treat MIH [34]. 

The difficulty in anaesthetizing the MIH molars has been widely documented. The pulp is not adequately protected from external stimuli as hypomineralized enamel is a poor insulator, and the tooth becomes susceptible to hot and cold conditions as a result. Chronic pulp stress causes an inflammatory reaction inside the pulp and pH changes at the periapical tissue level, resulting in hypersensitive pulp nerve tissue that activates with less stimulation than is typically required [29]. Multiple solutions have been proposed in the literature to address this problem. Several studies have suggested inhalation sedation as a way to enhance the pain threshold during dental treatment. Intraligamental, intraosseous, and palatal anesthesia are also further options. Studies comparing the effectiveness of the different types of local anesthesia (LA) agents in inferior alveolar nerve block found that none were significantly more effective than the others. In contrast, studies comparing their effectiveness in infiltration anesthesia revealed that articaine was significantly more effective. Providing dental treatment under rubber dam isolation can avoid sensitivity from other teeth, and using a saliva ejector instead of high-volume suction for hypersensitive teeth could be a more comfortable choice [1,29].

### 7.2. Treatment of Anterior Teeth

Children’s teeth that are discolored might have a significant psychological impact. Treatment of MIH has been demonstrated to enhance children’s overall health and OHRQoL in relation to their dental health. Children require a careful approach due to their large pulp chambers, high pulp horns, and underdeveloped gingivae. In addition, a minimally invasive technique preserves the tooth structure for future restorative alternatives. Children with poor oral hygiene, cariogenic diets, and numerous carious teeth should postpone their cosmetic treatment until an improvement has been proven and the carious teeth are addressed. There are few research studies available on MIH-affected incisors, and the success rates reported in these studies are inconsistent. As a result, no advice for a specific technique can be offered. A combination of approaches could be required due to the variability in the opacities and discoloration [7,23,35].

#### 7.2.1. Microabrasion

Microabrasion, using 18% hydrochloric acid or 37% phosphoric acid, followed by a CPP-ACP remineralizing agent, appears to be effective in enhancing the cosmetic appearances of whitish creamy opacities. It is feasible to use silicon carbide abrasive paste or pumice slurry. This is a minimally invasive procedure that only removes the top 100–200 m of enamel when applied correctly. Therefore, it is not ideal for opacities of greater depth [23,36].

#### 7.2.2. Resin Infiltration

For all kinds of opacities, resin infiltration with an etchant of 15–20% hydrochloric acid, ethanol, and TEGDMA monomer has been recommended. The damaged incisors can be given a simple and less invasive surgery to improve their translucency, optical properties, and overall color. Since infiltrated enamel is more prone to discoloration, improved oral hygiene practices are therefore important [23,35].

#### 7.2.3. The Etch-Bleach-Seal Technique

Although its effectiveness has been questioned in MIH, the etch-bleach-seal procedure is a less invasive technique for removing yellow-brown stains. The tooth is bleached for up to twenty minutes with 5% sodium hypochlorite, then etched with 37% phosphoric acid, and sealed with clear resin [7,23].

#### 7.2.4. External Bleaching

External bleaching is another non-invasive option for adolescents who want to mask their white opacities by making the teeth whiter overall. It comes in the form of hydrogen peroxide (up to 6%) or carbamide peroxide (10% or 16%) gels utilized in custom-made trays. Gingival irritation and sensitivity are common side effects that should be taken seriously, especially in young children [7,37].

#### 7.2.5. Composite Restorations or Veneers

Composite restorations can hide opacities of all colors and replace spots where enamel has broken down, with or without the removal of enamel. However, deeper opacities may necessitate enamel removal, and because of the pulp anatomy of young incisors, this should be performed as carefully as possible. An opaquer may be necessary before the composite is applied to cover any yellow-brown discoloration without removing too much enamel [23]. Composite veneers, on the other hand, are a more conservative method as they can be performed without tooth preparation, that is, without even removing the enamel of defective teeth. These treatments may be recommended for larger enamel defects caused by exposed dentin or broken enamel [35]. 

#### 7.2.6. Porcelain Veneers

This treatment line is recommended for patients above the age of 18 who have developed a matured gingival margin. This technique could be considered as an alternative when other options have failed to provide satisfactory results [35].

#### 7.2.7. A Combination of Treatment Options

For MIH-affected anterior teeth, a combination of treatment options may be the best long-term solution. Microabrasion, resin infiltration, external bleaching, and composite restoration were all used in a recent study, with each participant receiving a customized treatment plan based on their clinical needs. The findings showed that straightforward, minimally invasive procedures can produce positive clinical and psychosocial outcomes. [38]. In addition, some case reports have shown the benefits of these combined treatment choices in MIH-affected anterior teeth [26,39]. To ascertain the effectiveness and long-term results of such combined treatment approaches in anterior teeth impacted by MIH, more research is required.

### 7.3. Treatment of Posterior Teeth

A preventive approach should be applied if applicable as it is considered the most conservative treatment, especially in mild MIH cases. However, in more extensive lesions atraumatic restorations, composite resin, indirect restorations, or prefabricated metal crowns may be needed [23]. 

#### 7.3.1. Atraumatic Restorations

Atraumatic restorations are usually indicated from uncooperative children or children who cannot reach dental care. This treatment approach is based on glass ionomer restoration which can prevent tooth hypersensitivity and enamel break-down. The advantages of the glass ionomer include being hydrophilic, thereby requiring minimal isolation in addition to the fluoride release and recharge properties. On the other hand, glass ionomer is soluble over time, making it an interim restoration in addition to its low mechanical properties, which excludes its use in high-stress areas [40]. However, hybrid and high-viscosity glass ionomers have shown promising results in terms of their longevity and mechanical properties [41]. 

#### 7.3.2. Composite Resin Restorations

Composite resin restorations are one of the preferable options in the treatment of MIH lesions if the area is properly isolated. The simple direct application with the easy repair of the restoration makes it an excellent choice to restore both the esthetics and function of the damaged teeth. However, care must be taken when preparing the cavity as it is recommended to remove the hypomineralized areas in the enamel before bonding the restoration; otherwise the composite will suffer from poor retention to the defective enamel and low-bond strength [42]. 

#### 7.3.3. Indirect Restorations

Indirect restorations have also been reported to be a successful option for the treatment of moderate and sometimes severe MIH lesions. They are usually utilized in cases with the involvement of multiple cusps or surfaces. Three types of indirect restorations are available: indirect resin composite, cast metal alloys, and ceramics. Compared to prefabricated metal crowns, it is a more conservative option with less irritation to the gingival tissues as the restoration is placed away from the gingiva. On the other hand, this technique specific cavity features, which may be technique sensitive and necessitate the removal of any hypomineralized tissues. In addition to the need for temporization between visits, long chair times and relatively high costs compared to direct restorations are also considered as disadvantages. Concerning the best material, metallic indirect restorations usually have a higher wear resistance compared to the resin composite, but also present poorer esthetics. Furthermore, the resin composite has the advantage of the ease in repairability. Ceramic indirect restorations have superior esthetics and high wear resistance, but also require more teeth preparation, which may be risky in preparing teeth with high pulp horns. Thus, no single material can be recommended over the other, and the choice usually depends on each case [40,43].

#### 7.3.4. Prefabricated Metal Crowns 

Compared to custom-made crowns, prefabricated metal crowns are considered as a cost-effective treatment option for patients with moderate or severe MIH, showing a high success rate. The advantages of these crowns include the full protection and isolation of the sensitive and weakened crown in only a single visit. These crowns also maintain occlusal and proximal contacts, thus preserving the patient’s arch integrity and vertical dimension. On the other hand, this method may affect the health of the periodontium by the increasing pocket depth [23,44]. Another interesting use of the prefabricated metal crowns in primary teeth when they are affected by hypomineralization, and caries is the Hall technique. This technique involves a minimally invasive approach by cementing the prefabricated metal crowns with the glass ionomer without the removal of the caries; thus no LA or tooth preparation is needed [23,45].

#### 7.3.5. Scheduled Extractions 

This type of treatment is reserved solely for extensively decayed teeth with pulpal involvement, abscess, or cellulitis. Nonetheless, the closure of the extraction space may not occur spontaneously and may instead require orthodontic space closure or the maintenance of the space for a future prosthesis. Consequently, it is critical to assess the presence of any malocclusion or hypodontia prior to choosing this treatment option. This is to ensure that such a treatment would not exacerbate any pre-existing concerns–often culminating in negative outcomes. Furthermore, the child’s developmental age must also be taken into account when making treatment decisions. Ultimately, it is crucial to decide whether extraction at this age or not would be the most beneficial [46,47].

### 7.4. Digital Workflow and Computer-Aided Design and Computer-Aided Manufacturing (CAD–CAM)

The use of intraoral scanners (IOS) has created a new opportunity for repairing MIH teeth, since it makes it easier to deal with the children’s uncooperative behavior and allows for the preservation of tooth structure and long-lasting restoration. Children with MIH can benefit from a revolutionary minimally invasive treatment strategy that uses the digital workflow with IOS and the CAD–CAM manufacture of the repair. Due to its excellent scanning accuracy, it offers permanent restorations to young patients [48]. 

## 8. Strengths and Limitations

### 8.1. Strengths

1. Comprehensive synthesis of evidence: the current literature review provides a comprehensive synthesis of the existing studies, bringing together evidence from multiple sources to provide a broader understanding of the etiology and management of MIH. This can help identify the common trends, patterns, and gaps in the current knowledge base;
2. Integration of diverse perspectives: our review encompassed studies from various geographical regions, different populations, and diverse methodologies. This integration allows for a more comprehensive understanding of the global prevalence, risk factors, and management strategies associated with MIH;
3. Consideration of multiple outcomes: the present literature review assessed various outcomes studied across different research studies, such as the diagnostic criteria, treatment modalities, and patient-reported outcomes. This consideration of multiple outcomes thereby provides a more holistic view of the etiology and management of MIH;
4. Educational resource: this literature review can serve as a valuable educational resource for dental professionals, researchers, and students. It can help disseminate knowledge, summarize the current understanding of MIH, and provide a foundation for evidence-based practice and further research.

### 8.2. Limitations

1. Limited research: MIH is a relatively new condition, and the understanding of its etiology and management is still evolving. As a result, there may be limited studies available on the topic, leading to a scarcity of high-quality evidence;
2. Heterogeneity of studies: the available research on MIH may vary in terms of the study design, sample size, diagnostic criteria, and outcomes measured. This heterogeneity can thereby make it challenging to compare and synthesize the results, potentially impacting the overall reliability and generalizability of the findings;
3. Publication bias: review articles are susceptible to publication bias, where positive or significant findings are more likely to be published than negative or nonsignificant findings. This bias can skew the overall conclusions of the review if studies with unfavorable results are underrepresented;
4. Lack of long-term studies: MIH requires long-term follow-up and management to assess the effectiveness of different treatment approaches. However, there may be a scarcity of long-term studies evaluating the outcomes of various interventions, which can thereby limit the comprehensive understanding of the condition.

## 9. Conclusions and Future Directions

The public health issue of MIH must be viewed as having painful, aesthetic, and detrimental effects on the OHRQoL of affected children. Although many studies have discussed the different options for managing MIH lesions, evidence-based studies to address the long-term outcomes are still lacking. Regarding the etiology of MIH, future clinical studies should be directed to evaluate the impacts of each systemic etiological factor and its different types of management on normal amelogenesis. Regarding early detection and diagnosis, educating general dentists and pediatric dentists about the early signs and symptoms of MIH, and encouraging them to perform routine dental examinations in children to detect MIH at its earliest stages are recommended. Future studies should be directed towards pre-eruption diagnosis and early approaches to prevent the post-eruption breakdown and caries. Regarding the treatment of MIH, a combination of preventive measures should be considered, such as resin infiltration, fluoride varnish application, and sealants, to minimize enamel breakdown and improve esthetics. Further research in the field should be directed towards enhancing adhesion and exploring novel materials and techniques, such as bioactive restorative materials and digital dentistry. In addition, the long-term success, psychosocial, and economic impacts of MIH treatments must be studied. 

## Figures and Tables

**Table 1:** Summary of the potential risk factors associated with MIH.

Prenatal Stressors	Perinatal Stressors	Postnatal Stressors
- Medication - Smoking - illness - Stress - Hypocalcemia - Diabetes mellitus	- Cesarean delivery - Birth complication - Low birth weight - Breast milk contamination with an herbicide, such as dioxin	Conditions: - Asthma - Fever - Chicken pox - Measles - Rubella, - Early childhood caries Medications: - Amoxicillin - Antiepileptic - Antiasthma - Chemotherapeutic
**Systemic disease**	**Environmental**	**Genetic variant**
- Epilepsy - Respiratory diseases, such as pneumonia - Cyanosis - Vitamin D deficiency - Allergy - Tonsillitis - Otitis media - GIT disease	- Hypoxia - Disorders of calcium and phosphorus - Malnutrition - Exposure to bisphenol A	- SCUBE1 - MMP–20 - AMBN - ENAM - AMELX

**Table 2:** Description of the severity level according to the EAPD criteria [23].

Severity Level	Signs and Symptoms
Mild	Distinct enamel opacities that do not cause enamel degradation Induced sensitivity to outside stimuli, such as air and water, but not to brushing Mild aesthetic issues regarding the incisors’ discoloration
Severe	Clearly defined enamel opacities with caries and disintegration Spontaneous and enduring hypersensitivity impacting function, such as mastication and brushing Strong concerns about aesthetics that could affect sociopsychology

## Data Availability

Data created or analyzed during this study is available upon request from the corresponding author.
